# Educational and Behavioral Counseling in a Methadone Maintenance Treatment Program in China: A Randomized Controlled Trial

**DOI:** 10.3389/fpsyt.2018.00113

**Published:** 2018-04-04

**Authors:** Pulin Liu, Ranran Song, Yao Zhang, Cheng Liu, Bingxi Cai, Xuebing Liu, Jiaoyuan Li, Xueqin Chen, Juntao Ke, Jiao Lou, Wei Chen, Beibei Zhu, Li Zou, Yang Yang, Ying Zhu, Yajie Gong, Rong Zhong, Xiaoping Miao

**Affiliations:** ^1^Department of Epidemiology and Biostatistics, Ministry of Education and Ministry of Environmental Protection, State Key Laboratory of Environmental Health (Incubation), School of Public Health, Tongji Medical College, Huazhong University of Science and Technology, Wuhan, China; ^2^Wuhan Centers for Disease Prevention and Control, Wuhan, China; ^3^Wuhan Mental Health Center, Tongji Medical College, Huazhong University of Science and Technology, Wuhan, China

**Keywords:** methadone maintenance treatment, counseling, psychosocial intervention, randomized controlled trial, China

## Abstract

**Introduction:**

Methadone maintenance treatment (MMT) programs have been rapidly scaled up nationwide in China in recent years, and psychosocial intervention measures, including counseling, were recommended for improving the outcomes of MMT. However, the effectiveness of counseling in MMT programs remains controversial. This study investigated the efficacy of educational and behavioral counseling (EBC) mode in an MMT program in China.

**Methods:**

A total of 125 eligible participants were randomized to EBC or a control group. Patients in the EBC group received weekly, manual-guided, group educational counseling for 8 weeks and individual behavioral counseling for the next 8 weeks. Patients in the control group received standard methadone maintenance treatment as usual (TAU).

**Results:**

During the 16-week trial, the EBC group showed better treatment attendance (*P* = 0.022) and a greater increase in knowledge regarding heroin addiction (*P* = 0.001) and MMT (*P* = 0.005) than did the TAU group. Between the two groups, there were no significant differences regarding drug abstinence and reduction of risky behaviors.

**Conclusion:**

EBC affiliated with MMT improved patients’ cognition and adherence to treatment, facilitating their successful recovery.

**Clinical Trial Registration:**

ChiCTR-IOR-15006673: http://www.chictr.org.cn.

## Introduction

China now has the world’s largest number of illicit drugs users (1, 2), with this population representing a major driver of the country’s HIV epidemic due to unsafe practices, such as sharing drug injection equipment (3, 4). As an apparently effective harm reduction measure for reducing illicit opiate use, drug-related criminal behaviors and risk of HIV (5–7), a national methadone maintenance treatment (MMT) program was initiated as an HIV control strategy in China in 2004 (4, 8). The program has seen a dramatic expansion from just 8 pilot clinics covering 1,029 clients in 2004 (8) to 763 clinics serving 412,686 total participants nationwide by the end of 2013 (9).

Despite the strong progress made in slowing the HIV infection rate among drug users in China, rapidly scaling up the MMT program has created a wide range of challenges and gaps that need to be addressed, such as the demand for qualified service providers, misconceptions regarding methadone medication, low average methadone doses, high rates of concurrent drug use among clients, high drop-out rates, and poor service quality in the clinics (9–12). Hepatitis C infection among drug users is another serious public health problem that needs to be addressed in China (2, 13–16). A recent study of high rate of hepatitis C seroconversion in MMT programs also indicated an urgent need to improve MMT treatment services in order to obtain maximum benefits from this harm reduction approach (17).

Previous research in Western countries has demonstrated that the provision of additional counseling, medical, and psychosocial services produced dramatic enhancements to the efficacy of treatment with methadone medication alone and could improve the outcomes associated with MMT (5, 7). Conversely, there is also evidence that adding any psychosocial support to standard maintenance treatment does not add additional benefits (18).

China’s national guidelines underline that comprehensive services, including behavioral interventions and psychological counseling, should play an important role in improving the efficacy of MMT programs (9). However, the guidelines do not provide a specific manual or existing model which can be applied to meet the psychosocial needs of MMT participants. Several previous randomized controlled trial researches in China has evaluated the availability and effectiveness of additional counseling or other psychosocial intervention measures, such as contingency management and cognitive behavioral therapy (19–22), but most of these studies focused on individual level interventions for newly admitted patients in MMT clinics. In the context of MMT-related misconceptions among participants and insufficient human resources among MMT service providers in China (9, 11, 23, 24), a combination of group counseling with individual counseling could require a compromise between affordable psychosocial services from providers and the varied requirements of MMT participants.

In this study, we provided educational and behavioral counseling (EBC), including group and individual modes, directed at the characteristics of MMT patients. We hypothesized that EBC in addition to MMT would have greater efficacy compared to the current MMT in improving treatment attendance and reducing drug use. A 16-week randomized controlled trial was designed to evaluate the efficacy of EBC among MMT patients, and such outcomes as treatment attendance, drug abstinence, knowledge related to MMT and reduction of risky behaviors were investigated and assessed.

## Methods

### Study Site and Participants

The study was conducted in Wuhan city, which is located in the central area of China and which initiated an MMT program in 2006. There were more than 20 MMT clinics serving over 13,000 opioid dependent patients in Wuhan. In consideration of the number of active patients, two of the largest MMT clinics affiliated with a mental health hospital were selected based on their convenience and willingness.

Study enrollment began in May 2012 and ended in July 2014. Candidate participants were recruited by the clinicians and the research assistant based on inclusion and exclusion criteria. Inclusion criteria were outpatients aged 18 to 65 years, registered as local residents met the Chinese Classification of Mental Disorders 3 criteria for opioid dependence, had a positive urine test result for opioid at least once within the last month, and had no contra-indications for taking methadone. Exclusion criteria included being dependent on alcohol, benzodiazepines, or sedatives, and being psychotic, having major depression, or having other life-threatening or unstable medical problems. An interview was arranged by the clinic staff if a candidate participant expressed interest in the study, and a trained research assistant would make an initial face-to-face screening and explain the goals and procedures of the study to potential participants.

### Sample Size and Randomization

The proposed sample size was estimated based on an approximate simple formula (25). According to the literature (9, 10, 26), treatment retention rate during study period in control groups was assumed to be 60–65% and the detectable between-group difference was pre-estimated at 20–25% after intervention. At a significance level of 0.05 (two-sided hypotheses) and power of 80%, we calculated that a minimum sample size of 54 subjects per group would be required. To compensate for participant drop-out, we decided to increase the target sample size to at least 60 subjects per group. A computer-generated randomization sequence assigned the eligible patients in a 1:1 ratio to the EBC or treatment as usual (TAU) group. Important variables, including gender, age, education, injection drug use, and years of MMT, were considered in order to ensure baseline comparability of the intervention group and control group.

### Assignment of Treatment

All patients in the clinics received methadone treatment according to the standard dosing protocols in the MMT programs in Wuhan. The initial dose of methadone was approximately 30–40 mg per day, and subsequently the methadone dose was increased by 10 mg increments every 2 days, and as long as there were no complaints of sedation or side effects, to a target dose of 60–120 mg daily. All doses were dispensed at the clinic under the direct observation of the clinic staff without take-home doses. All patients paid RMB 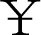
10 (USD $1.5) per dose regardless of the methadone volume, and in general, patients could consult doctors or nurses about their health concerns and clinics also offered some health education materials or gave an unscheduled educational lesson once a month or less.

Participants in the study were randomly allocated to receive one of the two treatments: the standard TAU or weekly EBC in addition to the standard treatment. All patients in the TAU group only received treatment services provided by clinic staff as usual. All participants in the EBC group received weekly EBC for 16 continuous weeks.

Educational and behavioral counseling intervention was made up of 16 counseling sessions (8 educational sessions and 8 behavioral sessions), and each session lasted for approximately 30–45 min. The educational sessions were conducted in a group of 3–4 persons in the first 8 weeks. These sessions included developing an understanding of opiate dependence as a chronic medical condition; the function of the methadone medication; the effect of heroin on the human body; the difficulty of opioids abstinence; the role of medication and counseling in the treatment process; basic knowledge of HIV/AIDS; as well as of HCV, HBV, and other blood-borne diseases; transmission and prevention of these diseases; and high-risk behaviors.

The behavioral sessions were conducted individually during the 9th–16th weeks and focused on behavioral improvement including how to distinguish the triggers related to opiate dependence, how to cope with peer pressure, how to learn relapse prevention skills, how to establish and strengthen self-efficiency and training to avoid risk behaviors associated with injection drug use or sexual activity. Counselors emphasized the positive consequences of behavioral change (for instance, the benefits of not using drugs and of a healthy lifestyle) rather than the negative consequences or dangers of continuing drug use. Counselors acknowledged the patient’s efforts and even their partial success at behavioral changes rather than focusing on the patient’s failures to accomplish treatment goals.

All of the EBC sessions were manual-guided, and PowerPoint presentations were facilitated by a mixture of bachelor’s- and master’s-level counselors, who had minimal prior experiences in drug use counseling. All counselors initiated counseling after completing training in EBC, including a multiday didactic workshop, case conferences, and weekly supervised sessions.

During the 16-week study period, all participants were interviewed and asked to fill out questionnaires during the initial session and at every 4-week evaluation session. Every participant was offered RMB 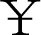
30 (USD $4.5) for transportation vouchers to help to attend each scheduled assessment appointment.

### Outcome Measures

All participants’ baseline demographic characteristics and daily methadone medication doses were recorded by MMT clinicians who had been trained by our research assistants. Questionnaires on knowledge and substance use characteristics, which consisted of 39 multiple-choice and true/false questions, were administered at baseline and every 4 weeks after study enrollment. These questionnaires were tested and adapted in MMT patients before the formal study. Illicit-drug use and opiate abstinence during treatment was measured by weekly urinalysis under supervision, using a semi-quantitative homogenous enzyme immunoassay for opioids with a cutoff set at 300 ng/ml.

The primary outcome measures were defined before the study began and included treatment attendance and drug abstinence. Treatment attendance was defined as days of participants taking methadone treatment during the 16-week trial. Drug abstinence was defined as the number and percentage of opioid-negative urine specimens during the 16-week trial. These two primary outcomes were collected objectively from clinic medication records.

The secondary outcome measures included an improvement of scores on the knowledge of HIV/AIDS, heroin addiction and MMT, and reductions in risk behaviors which included the frequencies of drug injection, needles, or other injection equipment sharing. These secondary outcomes were collected from scheduled questionnaires and participant self-report.

### Data Analysis

Data from all randomized participants were included. All analyses were based on the intention-to-treat principle and performed by IBM SPSS Statistics 19.0. First, the participants’ sociodemographic indicators and clinical characteristics at enrollment were compared between the two groups with the use of the Chi-square test for categorical data, and independent-sample *T*-test or Mann–Whitney *U* test were applied for continuous measures depending on the distribution. Second, the baseline characteristics were compared between participants who completed the study and participants who dropped out. Third, primary and secondary outcomes over time were evaluated between the EBC group and the TAU group, and generalized estimating equation (GEE) models were applied for further analysis. In the main outcomes analysis, group effects (EBC group versus TAU group) and time by group interactions were estimated. For continuous outcome variables (days of treatment attendance, knowledge scores), a linear GEE model was applied reporting unstandardized regression coefficients. For the categorical outcome variables (urine test result, risky behaviors), a binary or ordinal logistic GEE model was chosen reporting odds ratios. For all analyses, *P* values for two-tailed tests were reported. Significance level was set to 0.05.

## Results

### Recruitment of Participants

**Figure 1:**
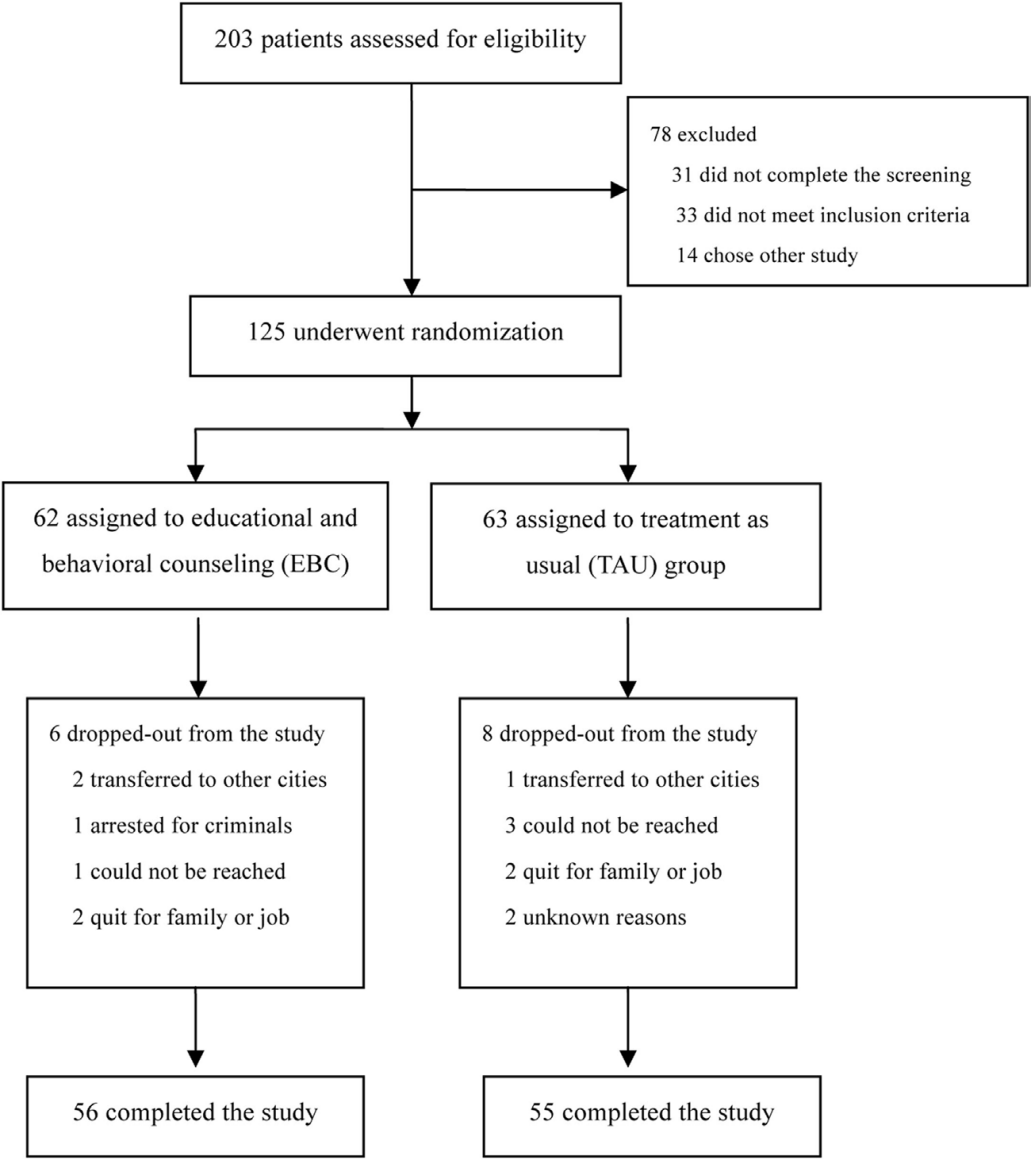
Enrollment and randomization.

Two hundred three patients expressed interest in study participation. However, only 172 patients completed the screening process while 31 patients did not show up at the screening interview. During the screening process, 33 patients were excluded due to not meeting the inclusion criteria, and 14 patients had already been enrolled in other studies. In total, 125 patients were enrolled and randomized to the study treatment period. Study completion was defined as not missing medication for more than seven consecutive days or not missing three or more counseling sessions. Fourteen patients did not complete the entire study for various reasons, including being transferred to other cities (3 cases), being arrested (1 case), being lost to follow-up (4 cases), or withdrawal due to family or job issues (4 cases). There were also unknown reasons (2 cases). Finally, 56 patients from the intervention group and 55 patients from the control group completed the whole study. A flow diagram of the study is shown in Figure 1.

### Demographic and Clinical Characteristics

The baseline demographic and clinical characteristics of the patients enrolled are provided in Table 1. The mean age of participants was 43.34 years (SD: 6.88). Most of participants were male (74.4%), married (42.4%), unemployed (72.8%), and had less than a high school education (95.2%). The majority of participants injected drugs before enrollment and had detoxification experiences, 88.1 and 89.6%, respectively. The average dosage of methadone taken by participants during the study was 77.51 mg/day (SD: 33.91). These characteristics did not differ significantly between the EBC and TAU participants.

**Table 1:** Characteristics of participants in educational and behavioral counseling (EBC) and treatment as usual (TAU) groups.

Characteristics	EBC (*N* = 62)	TAU (*N* = 63)	χ^2^ or *t*	*P*-value
Gender, *n* (%)			0.128	0.721
Male	47 (75.8)	46 (73.0)		
Female	15 (24.2)	17 (27.0)		
Education level, *n* (%)			2.863	0.413
Primary school and less	5 (8.1)	4 (6.4)		
Junior middle school	40 (64.5)	36 (57.1)		
Senior high school	13 (21.0)	21 (33.3)		
College or higher	4 (6.4)	2 (3.2)		
Marital status, *n* (%)			1.079	0.898
Unmarried	17 (27.4)	14 (22.2)		
Married	26 (42.0)	27 (42.9)		
cohabitation	1 (1.6)	1 (1.6)		
divorced	16 (25.8)	20 (31.7)		
Widowhood	2 (3.2)	1 (1.6)		
Employment, *n* (%)			0.121	0.728
Yes	16 (25.8)	18 (28.6)		
No	46 (74.2)	45 (71.4)		
Injection history, *n* (%)			1.216	0.270
Yes	57 (91.9)	54 (85.7)		
No	5 (8.1)	9 (14.3)		
Detoxification history, *n* (%)			2.058	0.151
Yes	58 (93.5)	54 (85.7)		
No	4 (6.5)	9 (14.3)		
Age, mean (SD)	44.03 (7.44)	42.65 (6.26)	1.124	0.263
Drug use years, mean (SD)	17.03 (4.70)	15.35 (5.49)	1.840	0.068
Treatment years, mean (SD)	5.19 (1.85)	4.63 (1.85)	1.682	0.094
Methadone dosage (mg/day), mean (SD)	75.87 (36.01)	80.05 (33.89)	0.688	0.505

There were no significant differences on gender, education, the history of injection and detoxification, years of MMT, and methadone dosage comparing the patients who dropped out (14 cases) to patients who completed the study (111 cases). There were significant differences between these two groups in age, employment, and marital status (Table 2), indicating that young, unmarried, and unemployed patients were more likely to be unwilling to complete the study. The proportion of patients who had completed the study did not differ significantly between the EBC group (90.32%) and the TAU group (87.30%) (*P* = 0.592).

**Table 2:** Characteristics of participants and drop-outs.

Characteristics	Participants (*N* = 111)	Drop-outs (*N* = 14)	χ^2^ or *t*	*P*-value
Gender, *n* (%)			0.073	0.787
Male	83 (74.8)	10 (71.4)		
Female	28 (25.2)	4 (28.6)		
Education level, *n* (%)			5.615	0.132
Primary school and less	6 (5.4)	3 (21.4)		
Junior middle school	68 (61.3)	8 (57.1)		
Senior high school	32 (28.8)	2 (14.3)		
College or higher	5 (4.5)	1 (7.1)		
Marital status, *n* (%)			13.331	0.010*
Unmarried	22 (19.8)	9 (64.3)		
Married	50 (45.0)	3 (21.4)		
cohabitation	2 (1.8)	0 (0)		
divorced	34 (30.6)	2 (14.3)		
Widowhood	3 (2.7)	0 (0)		
Employment, *n* (%)			5.891	0.015*
Yes	34 (30.6)	0 (0)		
No	77 (69.4)	14 (100)		
Injection history, *n* (%)			0.261	0.609
Yes	98 (88.3)	13 (92.9)		
No	13 (11.7)	1 (7.1)		
Detoxification history, *n* (%)			0.179	0.672
Yes	99 (89.2)	13 (92.9)		
No	12 (10.8)	1 (7.1)		
Age, mean (SD)	43.86 (6.61)	39.14 (7.76)	2.469	0.015*
Drug use years, mean (SD)	16.50 (5.141)	13.71 (4.795)	1.921	0.057
Treatment years, mean (SD)	4.96 (1.892)	4.50 (1.653)	0.876	0.383
Methadone dosage (mg/day), mean (SD)	78.04 (35.07)	77.50 (34.54)	0.054	0.957

*P < 0.05.

An overview of the primary and secondary outcomes of the patients in the EBC and TAU groups was presented in Table 3. The results showed no significant differences in the proportion of opiate-negative urine specimens (*P* = 0.054), the proportion of patients who had no injection within the last month (*P* = 0.816), and proportion who had no needle or other equipment sharing (*P* = 0.986). Significant differences were found in treatment attendance (*P* = 0.025), total knowledge scores, and subgroup scores of knowledge related to heroin addiction and MMT (*P* < 0.001), which indicated that the EBC group had better performance on adherence to treatment and knowledge awareness improvement in general. Further statistical analysis of GEE models was implemented to control for drop-outs’ incomplete data and to correct within-subject correlations in repeated measurements.

**Table 3:** Primary and secondary outcomes in educational and behavioral counseling (EBC) and treatment as usual (TAU) groups.

Outcomes	EBC (*N* = 62)	TAU (*N* = 63)	*P*-value
**Primary outcomes**			
Treatment attendance-days			
Median	91.00	79.00	0.025*
IQR	73.75–102.25	47.00–99.00	
Opiate-negative urine specimens-%			
Mean	76.74	67.38	0.054
95%CI	70.75–82.47	60.11–74.17	
**Secondary outcomes**			
Total score of knowledge			
Mean	10.56	8.94	<0.001**
95%CI	10.25–10.87	8.61–9.27	
Subscore 1			
Mean	2.25	1.16	<0.001**
95%CI	2.11–2.40	1.03–1.29	
Subscore 2			
Mean	4.81	4.24	<0.001**
95%CI	4.66–4.97	4.03–4.44	
Subscore 3			
Mean	3.49	3.55	0.475
95%CI	3.39–3.60	3.44–3.65	
No Injection within last month-%			
Mean	52.9	57.4	0.816
95%CI	47.1–58.8	51.2–63.2	
Needles or other equipment Sharing-%			
Mean	13.9	13.2	0.986
95%CI	9.9–17.9	9.3–17.4	

*CI, confidence interval; IQR, inter-quartile range; Subscore1, score of knowledge related to heroin addiction; Subscore2, score of knowledge related to methadone maintenance treatment; Subscore3, score of knowledge related to HIV/AIDS*.

*P < 0.05.

**P < 0.01.

### Generalized Estimating Equation Analysis

**Figure 2:**
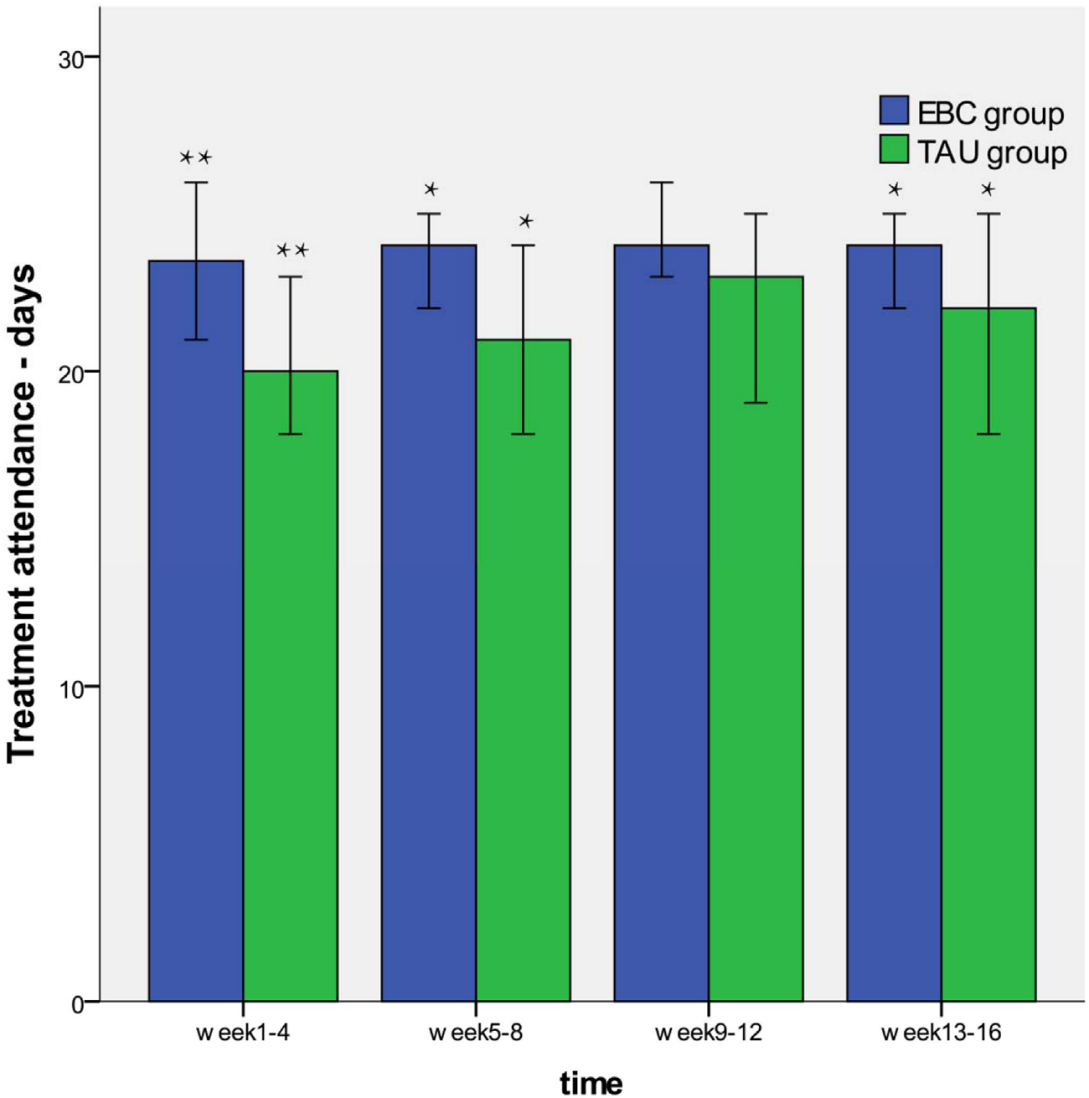
Treatment attendance of educational and behavioral counseling (EBC) and treatment as usual (TAU) group. The patients in the EBC group showed better treatment attendance than those in TAU group. There was not significantly group different during weeks 9–12 (*P < 0.05, **P < 0.01).

As shown in Table 4, the GEE analyses found that there was a significant difference in treatment attendance between the EBC and the TAU group (*P* = 0.022). However, the interaction effect of group and time was not significantly different (*P* = 0.480). These results indicated that patients in the EBC group had better treatment attendance than those in TAU group and the group difference did not differ as the time of assessment differed, and the characteristics also were presented in Figure 2.

**Table 4:** Generalized estimating equation analyses for primary and secondary outcomes (continuous variables).

Outcomes	Group	Estimated marginal means (SE)					Group effect	Time × group interaction
		Week 0	Week 1–4	Week 5–8	Week 9–12	Week 13–16	β (95%CI)	
Treatment attendance	EBC	–	20.11 (0.920)	22.41 (0.718)	22.25 (0.820)	22.13 (0.833)	3.089 (0.436–5.741)	wald = 2.475, *P* = 0.480
	TAU	–	18.71 (0.905)	19.72 (0.928)	19.84 (1.032)	19.04 (1.066)	wald = 5.208, *P* = 0.022*	
Total scores	EBC	8.39 (0.302)	10.75 (0.317)	10.87 (0.298)	11.26 (0.317)	11.6 (0.331)	2.294 (1.217–3.371)	wald = 26.829, *P* < 0.001**
	TAU	8.09 (0.328)	8.84 (0.332)	9.4 (0.312)	9.18 (0.441)	9.31 (0.438)	wald = 17.426, *P* < 0.001**	
Subscore1	EBC	1.36 (0.135)	2.46 (0.164)	2.36 (0.144)	2.42 (0.161)	2.70 (0.161)	1.341 (0.880–1.802)	wald = 19.986, *P* = 0.001**
	TAU	0.85 (0.113)	1.04 (0.126)	1.27 (0.142)	1.33 (0.170)	1.36 (0.171)	wald = 19.986, *P* = 0.001**	
Subscore2	EBC	3.98 (0.195)	4.79 (0.163)	4.96 (0.143)	5.21 (0.159)	5.17 (0.170)	0.884 (0.262–1.506)	wald = 9.632, *P* = 0.047*
	TAU	3.89 (0.195)	4.18 (0.230)	4.51 (0.194)	4.33 (0.254)	4.29 (0.268)	wald = 7.758, *P* = 0.005**	
Subscore3	EBC	3.05 (0.135)	3.50 (0.101)	3.55 (0.113)	3.64 (0.104)	3.74 (0.100)	0.069 (−0.264 to 0.403)	wald = 5.738, *P* = 0.220
	TAU	3.35 (0.132)	3.62 (0.087)	3.62 (0.101)	3.51 (0.142)	3.67 (0.137)	wald = 0.165, *P* = 0.684	

*CI, confidence interval; TAU, treatment as usual; EBC, educational and behavioral counseling; Subscore1, score of knowledge related to heroin addiction; Subscore2, score of knowledge related to methadone maintenance treatment; Subscore3, score of knowledge related to HIV/AIDS*.

*P < 0.05.

**P < 0.01.

**Figure 3:**
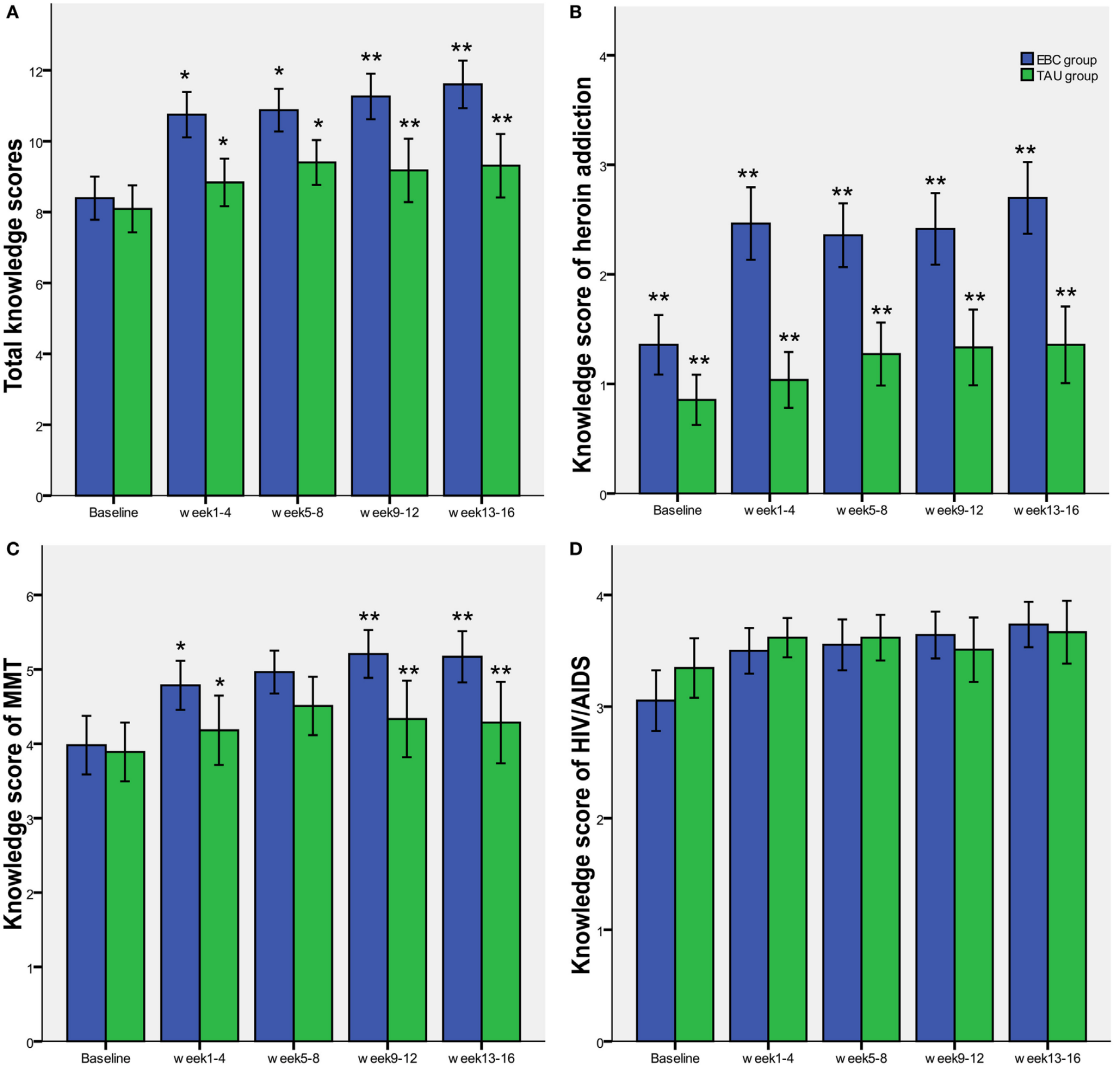
Knowledge scores of educational and behavioral counseling (EBC) and treatment as usual (TAU) group. **(A–C)** presented significant differences between the EBC and TAU group on the total knowledge scores and knowledge subgroup scores related to heroin addiction and methadone maintenance treatment, and **(D)** showed that there is no significant difference between the EBC and TAU group on the knowledge subgroup scores related to HIV/AIDS (*P < 0.05, **P < 0.01).

Regarding the total knowledge scores and knowledge subgroup scores related to heroin addiction and MMT, significant differences between the EBC and TAU group were found by the GEE analyses (*P* < 0.001, *P* = 0.001 and 0.005, respectively). There was also a significant interaction effect of group and time in these knowledge scores. These results demonstrated that EBC had a greater improvement on knowledge awareness than was seen in the TAU group, and this group difference became more apparent as the time of assessment differed (Figure 3). We did not identify a similar difference or time trend on the scores of knowledge related to HIV/AIDS between the EBC and the TAU group.

We found no significant differences between the EBC and the TAU group in the outcomes of the urinalysis results and reduction of risky behaviors, including drug injection frequency and sharing needles with others within the last month (Table 5). However, the interaction of group and time showed a significant difference in injection frequencies (*P* = 0.015). This implied that drug abstinence and reduction of risky behaviors did not have group differences, but injection frequencies were reduced as the time of assessment differed in both groups.

**Table 5:** Generalized estimating equation analyses for primary and secondary outcomes (categorical variables).

Outcomes	Group	OR	95%CI		Group effect		Time × group interaction	
			Lower	Upper	Wald Chi-square	*P*-value	Wald Chi-square	*P*-value
Urine analysis	EBC	0.423	0.100	1.791	1.365	0.243	18.179	0.253
	TAU	–						
Injection frequency	EBC	0.725	0.318	1.652	0.585	0.444	12.339	0.015*
	TAU	–						
Needles Sharing or not	EBC	0.444	0.100	1.976	1.136	0.287	7.431	0.115
	TAU	–						

*TAU, treatment as usual; EAU, educational and behavioral counseling*.

*P < 0.05.

## Discussion

In this study, we evaluated the efficacy of an EBC model, including group educational counseling sessions and individual behavioral counseling sessions, in an MMT program in China with a randomized control trial design. We observed that outcomes among patients in the EBC group, such as treatment attendance and scores of knowledge related to heroin addiction and MMT, differed significantly from those of the TAU group. We also observed that no significant group differences in the study phase were reported in the outcomes of opioids-negative urinalysis and reduction of risky behaviors. The findings of our study indicated that EBC intervention could contribute to a better understanding of treatment goals and to the improvement of treatment adherence, which reinforces the goal of continuously benefiting from MMT for opioids dependence patients. Our study results also demonstrated that current behavioral counseling may not be sufficiently effective to bring more significant behavioral change compared to a medication-only regimen and implied that more efforts beyond counseling should be made to help these patients.

Our study results are in contrast with other researchers who have demonstrated encouraging results for improvement in treatment retention and reduction of opiate use with ancillary psychosocial services in MMT patients in China. One pilot study involving 37 MMT patients showed that counseling services led by nurse counselors were effective in reducing HIV risk behaviors and drug use (19). Another randomized controlled trial evaluated the efficacy of a psychosocial intervention that included individual and family based counseling among newly admitted first-time MMT patients and observed a significant reduction in attrition and improvement in days of attendance (20). It should be noted that 79.2% (99/125) of the participants in our study had taken MMT for more than 4 years, and our findings demonstrate that the current EBC intervention has limited efficacy in these patients with many years of treatment. These findings cannot be simply interpreted to mean that methadone treatment is so efficacious that an EBC intervention adds little benefit and is not needed in the current MMT program at all. MMT providers in the participating clinics typically offer many additional services, such as regular communication on medication adherence, drug abstinence, and educational pamphlets, to all MMT patients (27). Many patients have benefited from these additional services and seen some successful progress after several years of medication treatment (28, 29). For these patients, a low-intensity EBC intervention would not be beneficial and a much higher intensity psychosocial intervention measurement is likely needed (30, 31). Next steps include developing tailored strategies for matching the diverse intensities of MMT service with the specific needs of individual patients and providing referrals to high intensity interventions as needed (32).

Psychosocial services, including counseling, in MMT in China have not been thoroughly developed (10, 28, 32, 33), and our present study could provide several lessons that could be used to improve the quality of MMT. We developed systematic educational materials, including PowerPoint slides and a manual for 16 consecutive counseling sessions. We also set up a procedure for training counselors to provide high quality counseling for MMT patients. These efforts and experiences make it possible to train more nurses or other MMT service providers who lack an advanced background in psychology to provide an EBC service in more clinics.

Our study had some potential limitations. First, we did not conduct a follow-up investigation to explore the long-term effects of the EBC, which limits the generalizability of our findings. Second, patients who enrolled in our study were not evaluated for degree of addiction severity, and patients with more severe addiction might have a stronger motivation or determination for change than those who were not enrolled. These parameters could be important confounding factors for assessing the effectiveness of intervention, and we should be cautious in applying the results to the general patient population. Third, we could not make more frequent urine measurements, and assessment of the use of illegal drugs other than opioids might have provided more meaningful information (34).

In summary, the results of our study have demonstrated preliminary feasibility and viability of EBC affiliated with MMT in China, and EBC has been demonstrated to contribute to a better improvement in knowledge awareness and treatment attendance. More research aimed at developing tailored strategies for various intensities of EBC is needed in order to help patients maintain abstinence and reduce individual risk behaviors.

## Ethics Statement

The study protocol was approved by the Human Investigation Committee of Tongji Medical School, Huazhong University of Science and Technology. Informed and voluntary written consent was obtained from every participant who passed the screening. This study was registered at http://www.chictr.org.cn (ChiCTR-IOR-15006673) on July 1, 2015, and the study methods and reporting were conducted in accordance with the CONSORT 2010 guidelines.

## Author Contributions

Conceived and designed the experiments: XM. Performed the experiments: PL, RS, YZhang, CL, BC, XL, JLi, XC, JK, JLou, WC, BZ, LZ, YY, YZhu, YG, and RZ. Analyzed the data: PL, RS, and BC. Contributed reagents/materials/analysis tools: PL and YZhang. Wrote the paper: PL, RS, and XM. Interpreted results and made many revisions of the manuscript: PL, RS, YZhang. Contributed to the revision of the manuscript and gave final approval for publication: PL, RS, and XM. All authors have reviewed and approved the manuscript as submitted.

## Conflict of Interest Statement

The authors declare that the research was conducted in the absence of any commercial or financial relationships that could be construed as a potential conflict of interest.
